# Assessment of Fetal Development Using Cardiac Valve Intervals

**DOI:** 10.3389/fphys.2017.00313

**Published:** 2017-05-17

**Authors:** Faezeh Marzbanrad, Ahsan H. Khandoker, Yoshitaka Kimura, Marimuthu Palaniswami, Gari D. Clifford

**Affiliations:** ^1^Department of Electrical and Computer Systems Engineering, Monash UniversityClayton, VIC, Australia; ^2^Electrical and Electronic Engineering Department, University of MelbourneMelbourne, VIC, Australia; ^3^Biomedical Engineering Department, Khalifa University of Science, Technology and ResearchAbu Dhabi, United Arab Emirates; ^4^Graduate School of Medicine, Tohoku UniversitySendai, Japan; ^5^Department of Biomedical Informatics, Emory UniversityAtlanta, GA, United States; ^6^Department of Biomedical Engineering, Georgia Institute of TechnologyAtlanta, GA, United States

**Keywords:** fetal development, gestational age, 1D Doppler ultrasound, cardiotocography (CTG), fetal electrocardiography (fECG), autonomic nervous system (ANS), fetal monitoring, systolic and diastolic time intervals

## Abstract

An automated method to assess the fetal physiological development is introduced which uses the component intervals between fetal cardiac valve timings and the Q-wave of fetal electrocardiogram (fECG). These intervals were estimated automatically from one-dimensional Doppler Ultrasound and noninvasive fECG. We hypothesize that the fetal growth can be estimated by the cardiac valve intervals. This hypothesis was evaluated by modeling the fetal development using the cardiac intervals and validating against the gold standard gestational age identified by Crown-Rump Length (CRL). Among the intervals, electromechanical delay time, isovolumic contraction time, ventricular filling time and their interactions were selected in a stepwise regression process that used gestational age as the target in a cohort of 57 fetuses. Compared with the gold standard age, the newly proposed regression model resulted in a mean absolute error of 3.8 weeks for all recordings and 2.7 weeks after excluding the low quality recordings. Since Fetal Heart Rate Variability (FHRV) has been proposed in the literature for assessing the fetal development, we compared the performance of gestational age estimation by our new valve-interval based method, vs. FHRV, while assuming the CRL as the gold standard. The valve interval-based method outperformed both the model based on FHRV. Results of evaluation for 30 abnormal cases showed that the new method is less affected by arrhythmias such as tachycardia and bradycardia compared to FHRV, however certain types of heart anomalies cause large errors (more than 10 weeks) with respect to the CRL-based gold standard age. Therefore, discrepancies between the regression based estimation and CRL age estimation could indicate the abnormalities. The cardiac valve intervals have been known to reflect the autonomic function. Therefore the new method potentially provides a novel approach for assessing the development of fetal autonomic nervous system, which may be growth curve independent.

## 1. Introduction

Estimation of the Gestational Age (GA) is crucial for antenatal diagnosis, monitoring fetal growth and detecting Intra-Uterine Growth Retardation (IUGR), predicting the delivery date and management of pre-term and post-term pregnancies, to ultimately prevent perinatal and neonatal mortality (Alexander et al., 1996; Taipale and Hiilesmaa, 2001; Bhutta et al., 2014; Chauhan et al., 2014). It is also a fundamental factor in ensuring the safety and effectiveness of medications during pregnancy (Reis and Källén, 2010; Andersen et al., 2013; Li et al., 2013). The GA has been traditionally estimated based on the Last Menstrual Period (LMP), which is the most affordable method specially for low and middle income countries (Wang et al., 2011; Deputy et al., 2017). However it is subject to human errors in recall or data entry, as well as biologically associated errors (Dietz et al., 2007; Lynch and Zhang, 2007). For example, the assumption of a regular 28-day menstrual cycle and ovulation on 14 days after the first day of LMP are not consistent and may vary from case to case (Dietz et al., 2007). As reported in the literature, the clinically estimated GA as collected on certificates of live birth based on prenatal and neonatal clinical assessments, exceeds the LMP-based GA by 2 weeks or more for more than 40% of the cases (Alexander et al., 1995). A recent study found the ovulation day as the most accurate predictor compared to LMP and ultrasound-based methods (Mahendru et al., 2016).

A more accurate and reliable estimation of the GA is provided through obstetric ultrasound which has been clinically established as the gold standard (Lynch and Zhang, 2007; Papageorghiou et al., 2014). A variety of sonographic measurements including Biparietal Diameter (BPD), Crown-Rump Length (CRL), Head Circumference (HC), Abdominal Circumference (AC) and Femur Length (FL) are used to estimate the GA (Hadlock et al., 1982; Dietz et al., 2007; Lynch and Zhang, 2007; Papageorghiou et al., 2014). Although these measures provide a more reliable estimation of GA compared to LMP, they are all based on physical growth (mass or proportions), which is affected by genetic variations (e.g., head size and shape in fetuses), gender and inherent variability in the fetal growth process (Hadlock et al., 1981; Sherwood et al., 2000; Lynch and Zhang, 2007; Kullinger et al., 2016). These methods may also systematically overestimate or underestimate the GA of the fetuses which are respectively large or small for GA (Sherwood et al., 2000; Lynch and Zhang, 2007). Unsuitable positioning of the fetus during measurement also causes error and the technique is subject to operator error, and the quality of the images (Hunter, 2009; Callen, 2011). For example, 95% confidence intervals of ±4 weeks were found for FL, which is one of the most accurate estimators. The prediction interval may be as large as ±7 weeks for the estimators with higher standard errors, such as AC (Sherwood et al., 2000). The error also increases with the gestational age and generally the ultrasound methods are more precise when performed in the first-trimester (Caughey et al., 2008; Falatah et al., 2014; Al-Amin et al., 2015). Pathological conditions may also introduce a high levels of inaccuracy or significant bias in many estimation methods.

Although in high income countries routine skilled ultrasound screening is performed, many factors limit its use in low income countries, including high cost of the equipment, lack of trained sonographers or physicians, as well as the skill required to perform a GA estimation test (Wang et al., 2011; McClure et al., 2014). We therefore propose an alternative technique to be used as an adjunct in estimating the GA where ultrasound imaging methods are unavailable or inadequate due to pathologies, unsuitable positioning, limited skills and technical issues.

One promising alternative GA estimator is Fetal Heart Rate (FHR) (Cha et al., 2001; Hoyer et al., 2013; Tetschke et al., 2016). FHR can be measured with affordable apparatus and little need for prior skill, and hence is a feasible approach in low income countries (Tezuka et al., 1998; Stroux et al., 2014). Early studies found a comparable accuracy of FHR-based method with CRL, in early pregnancy (38–64 days) (Tezuka et al., 1998), and the methods were further improved recently being more focused on neurological development (Hoyer et al., 2013; Tetschke et al., 2016). While ultrasound-based techniques are generally based on the physical development and influenced by genetic variations, FHR provides a marker for neuro-physiological development of the fetus, since it reflects the Autonomic Nervous System (ANS) control of the cardiovascular system, which matures through the progress in pregnancy. Various linear, nonlinear and complexity-based FHR variability (FHRV) parameters have been found to be closely related to the fetal development (Van Leeuwen et al., 2003; Hoyer et al., 2009; Wallwitz et al., 2012; Hoyer et al., 2013; Tetschke et al., 2016). Vagal and sympathetic activity rhythms and their interactions has been traditionally attributed to different frequencies of FHR fluctuations and their ratios (European Society of Cardiology, 1996) which can be evaluated during fetal development. More recently, a “functional Fetal Autonomic Brain Age Score” (fABAS) was introduced which leverages the FHR patterns in a multivariate analysis (Hoyer et al., 2013). However, FHR is influenced by arrhythmias, fetal behavioral/sleep states and heart rate patterns such as FHR accelerations and even maternal psychological and physiological conditions, particularly in mid- and late-gestation (Mantel et al., 1991; Monk et al., 2000; Ivanov et al., 2009; Marzbanrad et al., 2015b). These factors complicate the assessment of fetal development based on FHR.

Fetal cardiac valve intervals are alternative measures which could be obtained from non-invasive, low cost and easy-to-operate devices, and used as reliable markers for fetal development and well-being (Shakespeare et al., 2001; Khandoker et al., 2009; Marzbanrad et al., 2013b). These intervals are based on the opening and closing time of the fetal cardiac valves, namely the atrioventricular and semilunar valves. Automated techniques for estimation of these intervals from non-invasively recorded one-dimensional Doppler Ultrasound (1-D DUS) signal (conventionally used as FHR monitor) were proposed in our previous papers (Marzbanrad et al., 2013b, 2014a). The valve intervals can also be used to assess the ANS function, as an alternative to the FHRV, since the cardiac mechanics are known to reflect the autonomic control in the literature on adults (Berntson et al., 1994; Cacioppo et al., 1994a,b; Di Rienzo et al., 2013). As an example, the Pre-ejection Period (PEP), which is the interval from the onset of ventricular depolarization (the beginning of the QRS complex on the electrocardiogram) to the opening of aorta, reflects sympathetic influences on the heart (Cacioppo et al., 1994a; Mensah-Brown et al., 2010). In previous studies we found significant changes in the valve intervals with advancing GA (Marzbanrad et al., 2013a,b). In the work presented here we hypothesize that the fetal cardiac valve intervals, which are estimated automatically, can be used as a novel alternative measure of the GA, reflecting the physiological development of fetus.

## 2. Methods

### 2.1. Subjects and data acquisition

Doppler ultrasound and abdominal ECG signals were recorded simultaneously at Tohoku University Hospital, Japan, from 57 pregnant women with healthy single pregnancy who were not under any medication and 30 cases with fetal arrhythmia or abnormalities. The type of abnormalities and the GA for these 30 cases are presented in **Table 4** and more details about these arrhythmia and abnormalities can be found in Murray (2007), Allan et al. (2000), Allen et al. (2013), and Abuhamad and Chaoui (2012). All 87 fetuses had a GA of between 16 and 41 (32 ± 6) weeks at the time of recording. The GA was estimated using ultrasound imaging by a trained sonographer, by measuring fetal CRL at about 10 weeks, which is the length of embryos and fetuses from the head top (crown) to the bottom of the buttocks (rump). The study protocol was approved by Tohoku University Institutional Review Board and written informed consent was obtained from all participants. Table 1 summarizes the CRL-based gestational age, maternal age, weight and height for the healthy and abnormal cases.

**Table 1:** **Maternal age (years), height (cm) and weight (kg) as well as the CRL-based GA (weeks) for normal and abnormal groups are presented as mean ± standard deviation**.

**Group**	**Age (years)**	**Weight (kg)**	**Height (cm)**	**GA (weeks)**
Normal	26 ± 7	51.1 ± 5.7	152.7 ± 8.7	33 ± 6
Abnormal	35 ± 6	65.2 ± 12.1	162.6 ± 2.4	30 ± 6

The 1-D DUS signal was generated using a 1.5 MHz Corometrics 5700 Ultrasound transducer and the abdominal ECG signals were collected by a multichannel data acquisition system (fetal monitor 116, Corometrics Medical Systems Inc) with 1,000 Hz sampling frequency and 16 bit resolution. Twelve electrodes were used for abdominal ECG recordings, ten of which were arranged on the mother's abdomen, one reference electrode on the back and one electrode was set at the right thoracic position. The DUS transducer was placed on the lower abdomen and the audio output was connected to the input channel of the fetal monitor. All DUS and ECG recordings were 1 min in length and sampled at 1 kHz with 16-bit resolution. More details about the experimental set up can be found in Sato et al. (2007).

### 2.2. fECG extraction

Data from 12 channels were recorded bipolarly from the electrodes placed on the maternal abdomen, sampled every 1 ms (1 kHz sampling) with 16-bit resolution and bandpass filtered by 1–100 Hz finite impulse response filter. To separate fECG from the composite abdominal signal, a combination of maternal ECG cancellation and Blind Source Separation with a Reference (BSSR) was employed (Sato et al., 2007). In brief, electrical activities of the heart can be modeled as a vector in the direction of excitation, which is sometimes called the heart vector (Symonds et al., 2001). The maternal ECG component was excluded by subtracting the linear combination of mutually orthogonal projections of the heart vector. Subsequently, BSSR was used to extract fECG from complex mixture using DUS signal as a reference (Sato et al., 2007). Fetal QRS locations were detected by a modified Pan and Tompkins peak detection method as described in Behar et al. (2013).

### 2.3. Evaluation of data quality

The quality of 1-D DUS and fECG signals were assessed to exclude low quality signals and to evaluate the relationship of the final error in the GA estimation with the quality scores.

#### 2.3.1. fECG signal quality

A state of the art Signal Quality Index (SQI), known as “bSQI,” was used. This metric evaluates the agreement between two QRS detection methods with different robustness to noise (Clifford et al., 2012). The bSQI metric takes a range between 0 (lowest quality) and 1 (highest quality).

#### 2.3.2. 1-D DUS signal quality

Quality assessment of the DUS signal was performed using a method described in our earlier work (Marzbanrad et al., 2015a). The method is based on various quality indices of the high frequency component of the DUS signal. To isolate the high frequency component which is linked to the valves' movements, the DUS signal was decomposed by continuous wavelet analysis, as described in our earlier work (Khandoker et al., 2009; Marzbanrad et al., 2014a). Using a second order complex Gaussian as the mother wavelet, the signal at scale 2 (~200 Hz) was extracted and smoothed. The envelope of the absolute value of this signal was then estimated by interpolating the maxima and smoothing with a low pass filter. Each envelope was segmented into cardiac cycles using the corresponding RR intervals, estimated from fECG. The signal segments were then normalized by subtracting the mean and dividing by the standard deviation.

Twelve features were selected mainly based on the signal properties in the valve motion ranges compared to the remaining time intervals. The plausible valve motion ranges were defined as: Mc: (9–44), Ao: (45–90), Ac: (200–260), Mo: (265–326), all in msec following the segment onset (the preceding R-peak) (Khandoker et al., 2009; Marzbanrad et al., 2013b). The features selected were as follows and all were normalized to [0 − 1]:

- The ratio of the power (*SQI*_1_), number of peaks (*SQI*_2_), mean peak amplitude (*SQI*_3_) and variance (*SQI*_4_) in the valve motion range to the values in the remaining time intervals.
- kurtosis (*SQI*_5_), skewness (*SQI*_6_), Hjorth parameters (*SQI*_7_) and sample entropy (*SQI*_8_: *m* = 1, *r* = 0.1, *SQI*_9_: *m* = 1, *r* = 0.2, *SQI*_10_: *m* = 2, *r* = 0.1, *SQI*_11_: *m* = 2, *r* = 0.2), where *m* and *r* denote window length and tolerance, respectively.
- Minimum ratio of the 2nd to 1st singular value (*SQ*_12_) from Singular Value Decomposition (SVD) of a matrix containing consecutive windows of the signal with various sizes: 10, 15, 20,…,100.

An overall quality metric was obtained from the features *SQI*_1, 2,..,12_ using a Naïve Bayes (NB) classifier with kernel density estimate. The classifier was trained based on 345 cardiac cycles of the DUS signals which were annotated for quality by four independent annotators, as described in our previous paper (Marzbanrad et al., 2015a). The NB classifier uses the training data to estimate the conditional distribution of the features given the classes and also distribution of the classes. Then it estimates the posterior probability through the Bayes rule and classifies each sample to the most probable class. The same trained classifier was used in this study to classify the DUS quality. In our previous paper we used 10-fold cross validation and found the accuracy of 0.86 and 0.84 in train and test set, respectively.

### 2.4. Estimation of cardiac valve intervals

**Figure 1:**
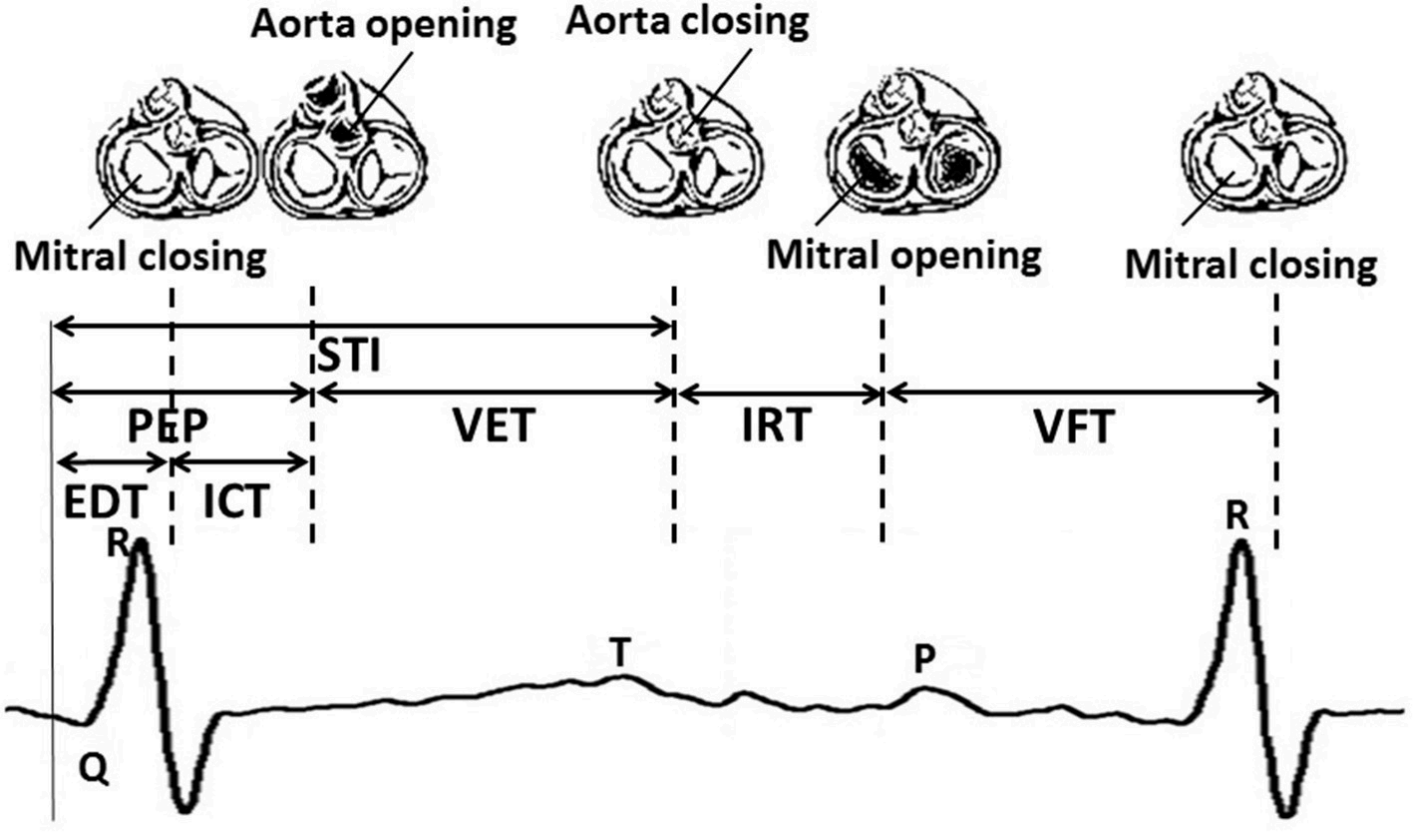
**An illustrative example of fetal cardiac intervals**. STI, Systolic Time Interval; EDT, Electromechanical Delay Time; ICT, Isovolumic Contraction Time; PEP, Pre-Ejection Period; VET, Ventricular Ejection Time; IRT, Isovolumic Relaxation Time; VFT, Ventricular Filling Time.

**Figure 2:**
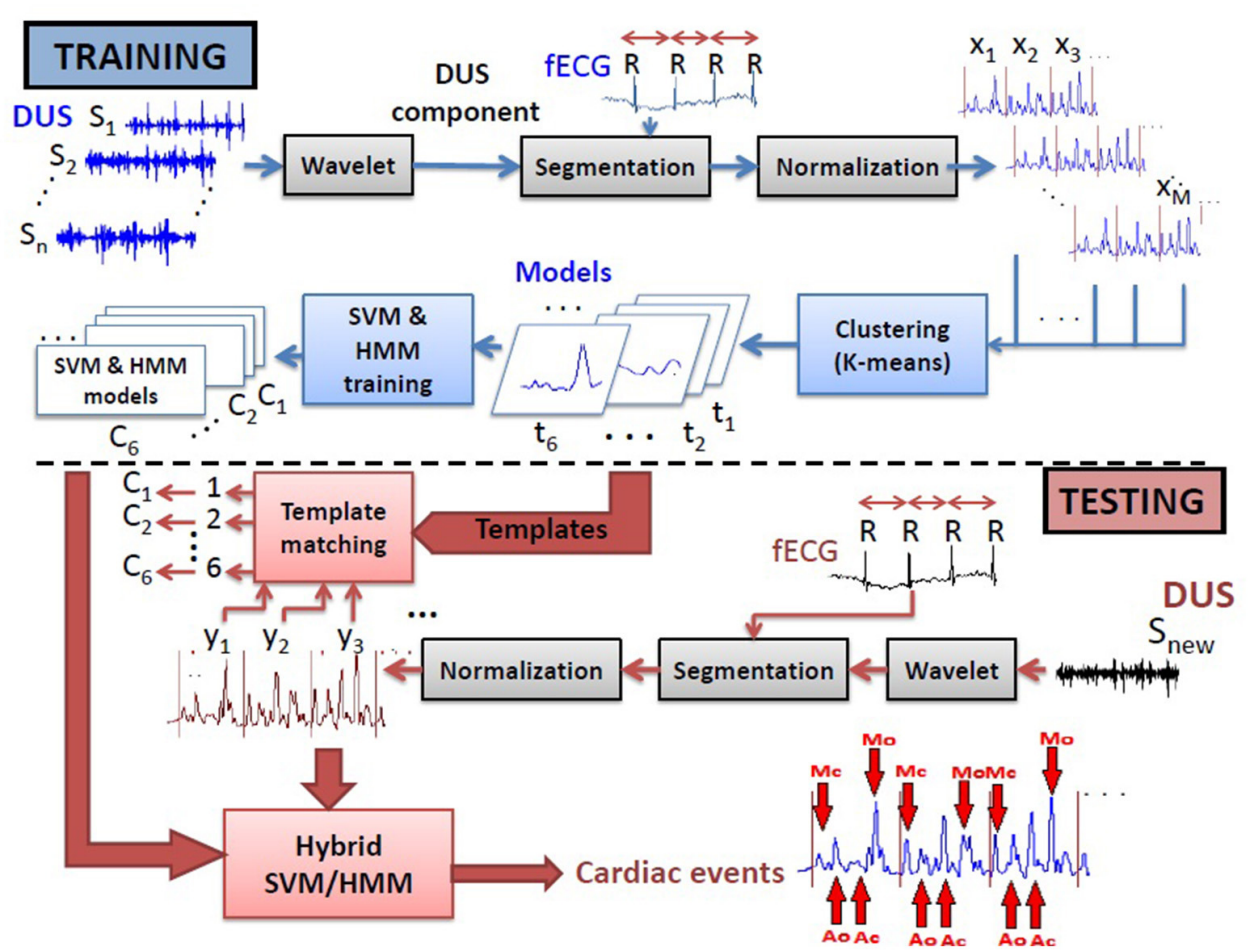
**Block diagram of the training and testing processes of the method used for automated identification of opening and closing of the valves (Marzbanrad et al., **2014a**)**.

The cardiac valve intervals are illustrated in Figure 1. These intervals were obtained based on the onset of the ORS complex detected as described above, and the opening and closing of the valves detected from the high frequency component of the DUS signal. The valve motion events were detected using a model-based method that was presented in our previous work (Marzbanrad et al., 2014a). This latter method is now summarized. The envelope of the high frequency component of the DUS signal, which were normalized and segmented into cardiac cycles (as described in Section 2.3.2), were clustered into six different patterns using K-means clustering. The key idea was to find the following events which correspond to the peaks of the high frequency components: Aortic valve opening (Ao), transitional event (T1), Aortic valve closing (Ac), transitional event (T2), Mitral opening (Mo), transitional event (T3), Mitral closing (Mc), transitional event (T4). The transitional events are related to extra peaks that do not correspond to any valve motion. A hybrid Support Vector Machine-Hidden Markov Model (SVM-HMM) was trained for each cluster separately, using the time (phase) and amplitude of the peaks of the signal as features, corresponding to one of the valve motion or transition events. The training and validation of this approach were based on expert annotation and simultaneous fetal echo-cardiography images, and were carried out in our earlier work (Marzbanrad et al., 2014a). To identify the events, each segment of the normalized envelope of the high frequency component was matched to the clusters that for which it had the minimum Euclidean distance to cluster's centroid. Then the sequence of events, which were attributed to the peaks of the signal, were identified by the Viterbi algorithm using the trained SVM-HMM specific to the corresponding cluster. The block diagram of this method is shown in Figure 2 and more details can be found in Marzbanrad et al. (2014a).

### 2.5. Estimation of the gestational age

Three sets of parameters were used to estimate the GA:

- Valve-timing parameters: From the parameters shown in Figure 1, Electromechanical Delay Time (EDT), Isovolumic Contraction Time(ICT), Ventricular Ejection Time (VET), Isovolumic Relaxation Time (IRT) and Ventricular Filling Time (VFT) were selected. Only Pre-Ejection Period (PEP) and Systolic Time Interval (STI) were excluded as they were linearly related to other intervals.
- FHR-related parameters: Time and frequency domain FHRV parameters were used including: Mean and standard deviation of RR intervals (mRR and SDRR), Root Mean Square of the Successive Differences (RMSSD) between adjacent RR intervals, low frequency (LF: 0.03–0.15 Hz) which is related to the neural sympathetic activity, medium frequency (MF: 0.15–0.5 Hz) corresponding to the fetal movements and maternal breathing and high frequency (HF: 0.5–1 Hz) which marks the presence of fetal breathing (typically present after 32nd week of gestation), the ratio LF/(MF+HF) and Total Power (TP). More details can be found in Signorini et al. (2003) and Van Leeuwen et al. (2003).
- Combined parameters: A combination of the FHR-related parameters and five valve timing parameters were used.

In order to estimate the GA from these parameters a stepwise regression analysis was employed based on individual and all combinations of parameters and the models including an intercept, linear, squared terms and cross-products. Stepwise regression automatically adds to or removes from the model in a forward and backward process to determine a final model, using an *F*-test applied to the sum of the squared error before and after adding a parameter (*p* < 0.05) as the criterion for including a parameter. Root Mean Squared Error, R-squared, adjusted R-squared and the *F*-test results vs. constant model were calculated for the regression of each set of parameters. An average leave-one-out cross-validation error in GA estimation was calculated. The difference between the CRL-based and regression-based GA estimate was made at every stage to provide an estimate of out of sample performance of the proposed approach.

The GA estimation error was compared for different parameters, including: FHR, fECG quality score and DUS signal quality score. The improvement of the GA estimation by applying threshold on the quality of the signals was also evaluated. Finally the optimal regression model which was obtained based on healthy cases was then used to estimate the GA of abnormal cases.

## 3. Results

### 3.1. Stepwise regression results

Using fetal heart valve timings as parameters, stepwise regression resulted in the following regression model for all healthy fetuses, without excluding the cases with low quality signals:

$$\begin{array}{l} \text{Estimated GA}=a_{0}+a_{1}EDT+a_{2}ICT+a_{3}VFT+a_{4}EDT\\ {}*ICT+a_{5}ICT*VFT \end{array}$$

where *a*_0_, *a*_1_, …, *a*_5_ are the coefficients. Table 2 shows the estimated coefficients and the Standard Error (SE). It shows the t-statistic for each coefficient to test the null hypothesis of the coefficient being zero, given the other estimators in the model. The *p*-value of the F-statistic for the hypothesis of the coefficient being zero is also shown.

**Table 2:** **Results of Stepwise regression using valve intervals, including estimated coefficients (***a***_**0**_, ***a***_**1**_, …, ***a***_**5**_) and Standard Error (SE) of the coefficients, t-statistic and ***p***-value for the F-statistic of the hypothesis of the coefficient being zero**.

	**Estimate**	***SE***	***t*-test**	***p*-value**
Intercept	−276.810	61.772	−4.481	4.218*10^−5^
EDT	5.496	1.215	4.525	3.641*10^−5^
ICT	7.897	1.743	4.530	3.574*10^−5^
VFT	0.682	0.267	2.551	0.014
EDT^*^ICT	−0.140	0.034	−4.142	1.295*10^−4^
ICT^*^VFT	−0.017	0.007	−2.273	0.027

The model was obtained based on the parameters in milliseconds and GA in weeks. Other results include: F-statistics: 15.1, p-value = 4.36^*^10^−9^, Standard deviation of the error distribution: 4.01 (weeks), R-squared: 60%, adjusted R-squared: 56%.

The statistics for the *F*-test on the regression model vs. constant model, showed significance of the model (F-statistics 15.1, *p*-value = 4.36 * 10^−9^). Standard deviation of the error distribution was 4.01 (weeks) and R-squared and adjusted R-squared were 60 and 56%, respectively.

FHRV parameters were also used to estimate the GA. The following regression model for all healthy fetuses, without excluding the cases with low quality signals, were obtained using stepwise regression:

$$\begin{array}{l} \text{Estimated GA}=b_{0}+b_{1}mRR+b_{2}SDRR \end{array}$$

where *b*_0_, *b*_1_, and *b*_2_ are the coefficients. Therefore only the mean and standard deviation of fetal RR-intervals significantly contributed to the model. Table 3 shows the estimated coefficients and SE of the coefficients. It shows the t-statistic for each coefficient to test the null hypothesis of the coefficient being zero, given the other estimators in the model. The *p*-value of the F-statistic for the hypothesis of the coefficient being zero is also shown.

**Table 3:** **Results of Stepwise regression using FHRV parameters, including estimated coefficients (***b***_**0**_, ***b***_**1**_ and ***b***_**2**_) and Standard Error (SE) of the coefficients, t-statistic and ***p***-value for the F-statistic of the hypothesis of the coefficient being zero**.

	**Estimate**	***SE***	***t*-test**	***p*-value**
Intercept	4.788	10.866	0.441	0.661
mRR	0.064	0.026	2.432	0.018
SDRR	0.120	0.058	2.044	0.046

*The model was obtained based on the parameters in milliseconds and GA in weeks. Other results include: F-statistics: 6.08, p-value: 0.004, standard deviation of the error distribution: 5.55 (weeks), R-squared: 18%, adjusted R-squared 15%*.

The statistics for the *F*-test on the regression model vs. constant model, showed significance of the model (F-statistics 6.08, *p*-value = 0.004). However the standard deviation of the error distribution was 5.55 (weeks) which was larger than the SD for the model with valve intervals, and R-squared and adjusted R-squared were only 18 and 15%, respectively, which were smaller than those of the model with valve intervals.

Using leave-one-out cross-validation, the mean absolute difference between the CRL-estimated GA and the GA estimated from the proposed model was found to be 5.1 weeks using FHRV parameters and 3.8 weeks using valve timing intervals and 4.2 weeks when all parameters combined. When attempting to select a combined model, none of the FHR parameters were selected and therefore did not provide any additional value or increase the GA estimation accuracy. In the leave-one-out process, a new regression model is obtained when excluding each case. Therefore the model from the combined parameters were not necessarily the same as the model obtained based on all cases, thus the mean absolute errors were different for the methods using valve intervals and combined parameters.

### 3.2. The effect of signal quality on GA estimation

The absolute error which was calculated using leave-one-out approach, was not significantly correlated with the quality scores while controlling for GA and FHR:

- Correlation with the fECG quality score: (*r* = −0.234, *p* = 0.088).
- Correlation with the DUS signal quality score: (*r* = −0.007, *p* = 0.958).

**Figure 3:**
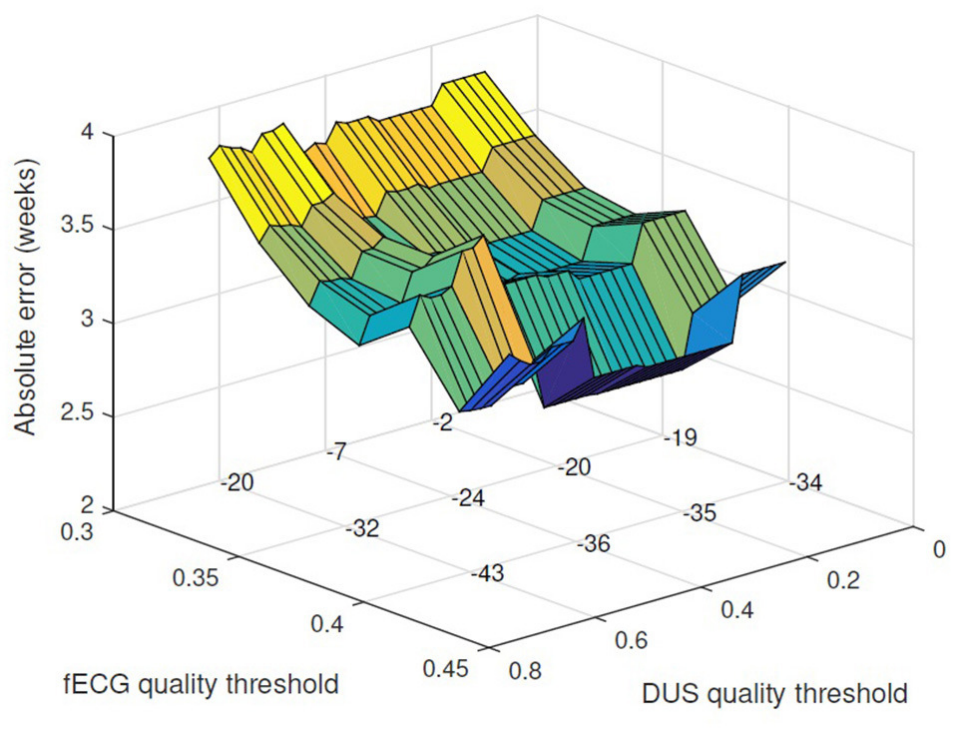
**Applying threshold for acceptable DUS and fECG signal quality, the absolute error for GA estimation is reduced**. The absolute error (weeks) is plotted vs. the thresholds for fECG and DUS signal quality scores. The number of excluded cases for different choices of the quality thresholds are shown on the grid knots.

**Figure 4:**
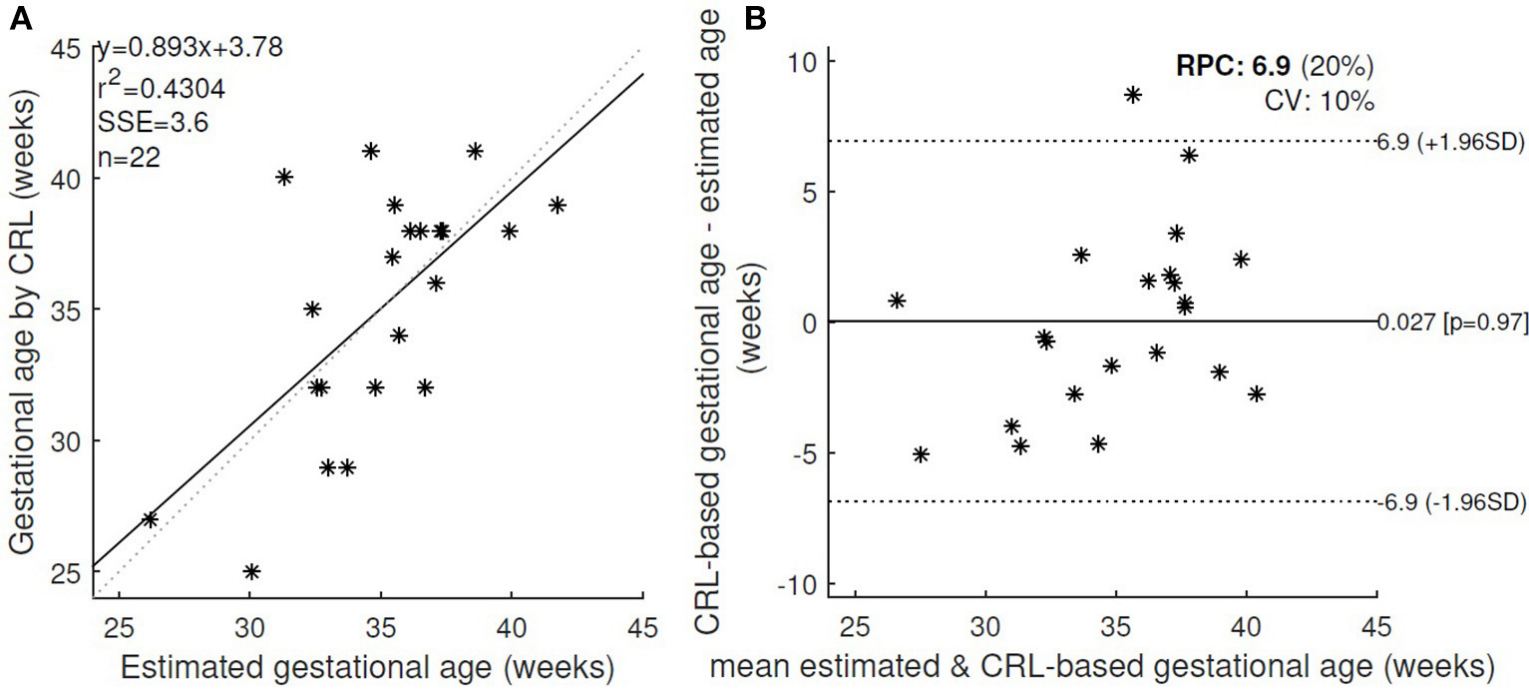
**(A)** The estimated GA using cardiac valve timings and the GA based on CRL as a gold standard were compared for 22 healthy fetuses with fECG quality score > 0.4 and DUS quality score > 0.3. r, Pearson correlation *r*-value; r2, Pearson *r*-value squared; SSE, sum of squared error; n, number of fetuses. **(B)** Bland-Altman plot (bias and 95% limits of agreement: 1.96 SD) for the estimated and CRL-based GA. RPC(%): reproducibility coefficient and % of mean values, CV: coefficient of variation (SD of mean values in %).

However the error was decreased by applying a threshold on the quality of the signals and excluding the cases with low quality score. Figure 3 shows the changes in the absolute error for various threshold values of fECG and DUS signal quality scores. Minimum absolute error 2.7 weeks was obtained when only the cases with fECG quality score > 0.4 and DUS quality score > 0.3 (22 cases in total) were considered. Applying higher thresholds would result in exclusion of more than 60% of cases. Figure 4 shows the estimated GA using cardiac valve timings compared to the GA based on CRL as a gold standard for 22 fetuses with fECG quality score > 0.4 and DUS quality score > 0.3. This figure also shows the 1.96 × SD of the error as the 95% limits of agreement between the estimated age by cardiac valve intervals and CRL.

Furthermore, the absolute error for estimating the GA based on the FHR-related parameters could be reduced to the minimum of 4.7 weeks by applying the threshold of 0.32 on the fECG quality.

### 3.3. Changes of the estimation error with GA and FHR

The correlation of the error (estimated GA using valve intervals - CRL-based GA) using leave-one-out cross-validation, with GA as well as FHR was calculated and the results are summarized as follows:

- The error of GA estimation based on the valve timings was inversely correlated with GA (*r* = −0.591, *p* < 0.001). Stronger correlation with GA was found (*r* = −0.654, *p* < 0.001) when it was controlled for other factors, such as FHR, and quality score for DUS and fECG. The GA was overestimated for the early gestation and underestimated for late gestation fetuses. The error was more significantly correlated with GA when the regression was based on FHR parameters (*r* = −0.939, *p* < 0.001).
- The absolute value of the error for estimation using valve timings was not significantly correlated with GA (*r* = −0.117, *p* = 0.385), nor was it significant while controlling for FHR and quality scores (*r* = −0.020, *p* = 0.884). The absolute error of the regression based on the FHR parameters was inversely correlated with GA (*r* = −0.422, *p* = 0.001), also when it was controlled for the quality scores (*r* = −0.446, *p* = 0.001).
- The error of GA estimation based on the valve timings was inversely correlated with FHR, when controlling for GA and quality scores (*r* = −0.325, *p* = 0.017). However, the absolute value of error was not significantly correlated to FHR (*r* = 0.1226, *p* = 0.3771).

### 3.4. Estimation of the GA for abnormal cases

The regression model based on the cardiac valve intervals of the healthy fetuses was used to estimate the GA of the fetuses with various abnormalities and arrhythmia. Table 4 shows the GA estimated by regression and CRL for each case.

**Table 4:** **Comparison of the estimated GA (weeks) using regression model based on the cardiac valve intervals with the gold standard (identified by CRL), for the cases with various types of anomalies and arrhythmias**.

**ID**	**CRL GA**	**Valve-based**		**FHR-based**		**FHR (bpm)**	**DUS/fECG SQI**	**Type of abnormality**
		**Est. GA**	**Error**	**Est. GA**	**Error**			
1	33	35	2	32	−1	202	0.8/0.5	Tachycardia
2	35	32	−3	29	−6	175	0.8/0.5	Tachycardia
3	38	33	−5	48^†^	10^*^	133	0.8/0.4	Arrhythmia
4	37	42	5	47^†^	10^*^	105	0.5/0.3	Bradycardia for SSS
5	38	41	3	76^†^	38^*^	104	0.6/0.3	Bradycardia for SSS
6	35	46^†^	11^*^	34	−1	138	0.8/0.4	WPW
7	37	37	0	118^†^	81^*^	117	0.9/0.3	PAC
8	32	36	4	33	1	142	0.7/0.4	Loss of FHRV-distress
9	30	36	6	33	3	149	0.4/0.4	Heart failure
10	33	33	0	34	1	132	0.1/0.5	Heart anomaly
11	36	33	−3	33	−3	145	0.9/0.5	Heart anomaly
12	30	32	2	35	5	135	0.3/0.3	Heart anomaly
13	34	35	1	30	−4	152	0.5/0.5	Heart anomaly
14	22	36	14^*^	41	19^*^	133	0.6/0.2	Heart anomaly
15	22	38	16^*^	54^†^	32^*^	122	0.6/0.3	Heart anomaly
16	36	32	−4	34	−2	144	0.9/0.3	Heart anomaly
17	28	29	1	32	4	147	0.7/0.3	TOF
18	28	30	2	43^†^	15^*^	145	0.5/0.3	TOF-VSD-PA-MS-PAC
19	23	70^†^	47^*^	63^†^	40^*^	67	0.5/0.2	VSD-ASD-CDH-CA
20	35	80^†^	45^*^	104^†^	69^*^	65	0.7/0.2	AV block
21	27	96^†^	69^*^	87^†^	60^*^	68	0.7/0.2	AV block-SA-CAV
22	24	82^†^	58^*^	86^†^	62^*^	62	0.6/0.3	PA-CAVC-SA-AV block-PS
23	26	35	9^*^	37	11^*^	127	0.3/0.3	Ebstein's anomaly
24	33	39	6	57^†^	24^*^	122	0.6/0.3	Cardiac dilatation-CHD
25	20	67^†^	47^*^	89^†^	69^*^	78	0.3/0.2	NIHF
26	29	35	6	33	4	144	0.9/0.3	NIHF-Hydrops amnii
27	18	25	7	29	11^*^	158	0.8/0.4	TTTS Donner
28	35	36	1	31	−4	152	0.3/0.5	Acute crisis-placental abruption
29	24	32	8	31	7	147	0.9/0.3	Placental dysfunction
30	31	20	−11^*^	30	−1	163	0.6/0.3	History of intrauterine death

† *Marks the estimated GA >42 weeks, where the abnormal condition affects the valve intervals or FHR, therefore the regression model fails to estimate the GA correctly*.

The difference (estimated GA - CRL GA) for the cases marked with ^*^ is outside the confidence interval of the error of GA estimation for healthy cases.

*SSS, Sick sinus syndrome; WPW, Wolff-Parkinson-White syndrome; PAC, Premature Atrial Contraction; TOF, Tetralogy of Fallot; VSD, Ventricular Septal Defect; PA, Pulmonary Atresia; MS, Mitral Stenosis; ASD, Atrial Septal Defect; CDH, Congenital Diaphragmatic Hernia; CA, Chromosomal Aberration; AV block, Atrioventricular block; SA, Single Atrium; CAV, Cardiac Allograft Vasculopathy; CAVC, Common Atrioventricular Canal; PS, Polysplenia Syndrome; CHD, Congenital Heart Disease; NIHF, Nonimmune Hydrops Fetalis; TTTS, Twin-to-Twin Transfusion Syndrome Donor (Allan et al., 2000; Murray, 2007; Abuhamad and Chaoui, 2012; Allen et al., 2013)*.

**Figure 5:**
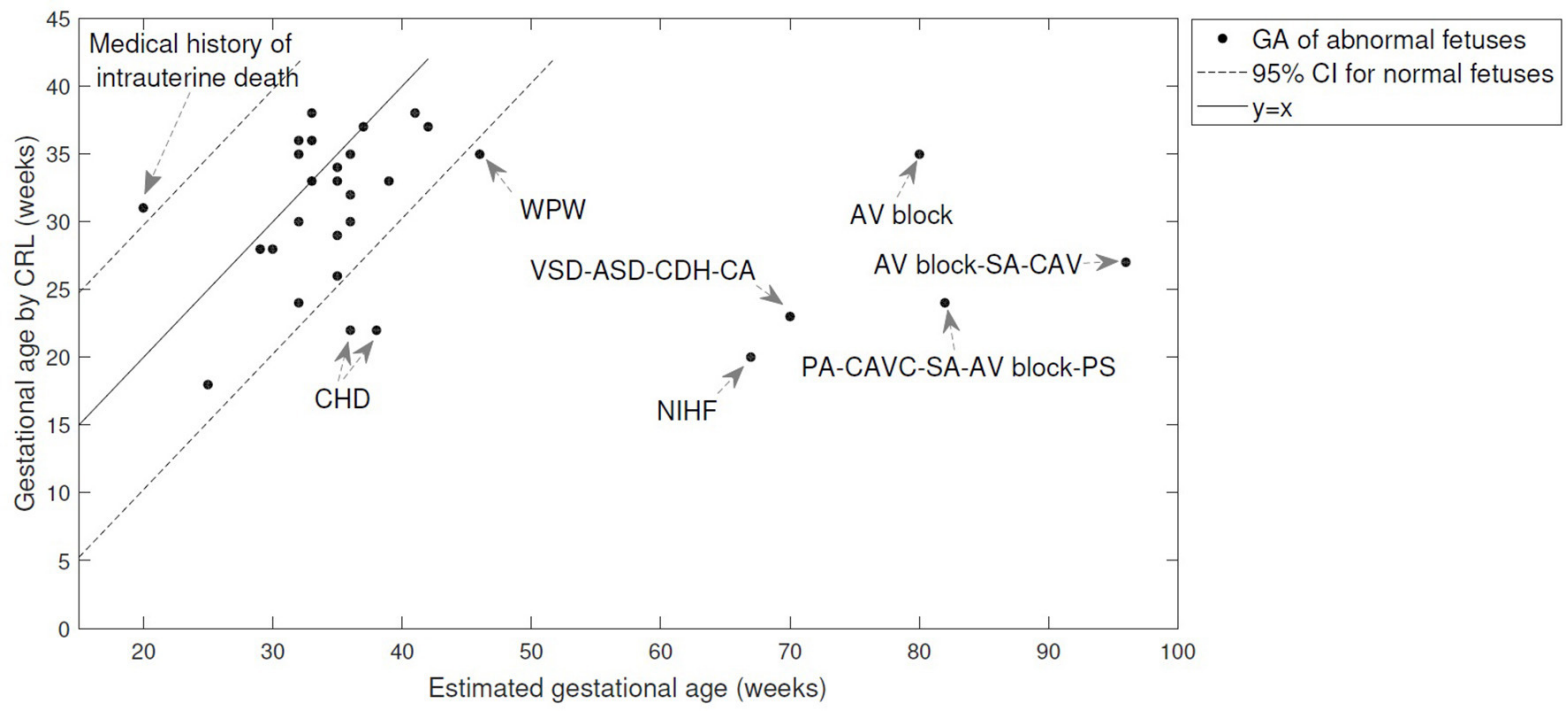
**The estimated GA using cardiac valve timings vs. the GA based on CRL as a gold standard are shown for 30 abnormal cases**. The 95% Confidence Interval (CI) for healthy cases (as shown in Figure 4) and *y* = *x* line are also shown for comparison. The abnormality types are specified for the cases with estimated GA being outside the 95% CI. More details can be found in Table 4. (WPW, Wolff-Parkinson-White syndrome; VSD, Ventricular Septal Defect; PA, Pulmonary Atresia; ASD, Atrial Septal Defect; CDH, Congenital Diaphragmatic Hernia; CA, Chromosomal Aberration; AV block, Atrioventricular block; SA, Single Atrium; CAV, Cardiac Allograft Vasculopathy; CAVC, Common Atrioventricular Canal; PS, Polysplenia Syndrome; CHD, Congenital Heart Disease; NIHF, Nonimmune Hydrops Fetalis).

The table shows that arrhythmia (for cases 1–5) results in 5 weeks or less error in estimating the GA using heart valve intervals, while the FHR-based model failed to estimate the GA for bradycardia and arrhythmia case. Figure 5 shows the estimated GA using cardiac valve timings vs. the GA based on CRL as a gold standard for the abnormal cases, compared to the 95% Confidence Interval (CI) for healthy cases (as shown in Figure 4). The specific abnormality types which resulted in estimated GA being outside the 95% CI are specified. The GA estimation using valve intervals clearly fails for some types of heart abnormalities such as ASD, VSD, SA and AV block (cases 19–22), due to their influence on opening and closing of the heart valves. FHR based model also failed for those anomalies as well as for the case with Premature Atrial Contraction (PAC) which was correctly estimated by the valve interval-based model.

## 4. Discussion and conclusion

In this paper a new approach is proposed for estimation of the GA using fetal cardiac valve intervals. These intervals were estimated by a fully automated method from the raw recordings, therefore is less affected by human errors compared to sonography or LMP methods. Furthermore, the apparatus used to obtain the valve intervals are easier to handle and require less skill to operate compared to standard sonography techniques. Although in this work we used non-invasive simultaneous recording of 1-D DUS signals and fECGs, the latter are only required for estimation of EDT which depends on the onset of QRS complex. It is also possible to use only 1-D DUS signals to obtain ICT, VET, IRT, and VFT using an automated technique that we proposed in previous work (Marzbanrad et al., 2013b). Since a 1-D DUS device can cost as little as $17 and can be performed by nonexperts with limited training, it can be used to estimate the GA in resource limited settings (Stroux et al., 2014).

Although we have not directly evaluated the ANS and its relationship with valve intervals, the complex interplay between autonomic control of the heart and cardiac mechanics characterized by the valve intervals, has been previously reported in literature and is consistent with the results of this study (Berntson et al., 1994; Cacioppo et al., 1994a,b; Di Rienzo et al., 2013). According to the studies on adult cases, PEP is attributed to the sympathetic nervous system effect on the heart (Cacioppo et al., 1994a). As shown in Figure 1, the PEP, which is the duration from Q-wave to aorta opening, is comprised of two intervals: the EDT, which is the Q-wave to mitral closing interval, and the ICT, which is mitral closing to aorta opening interval. Our results show that not only do the ICT and EDT contribute to the GA estimate but their interaction is also a significant contributor. According to the results of stepwise regression in Table 2, VFT was also selected as a contributing term to the estimate the GA. Although less emphasis has been placed on fetal VFT than other intervals, both in the literature and clinical practice, studies on adults found that VFT is controlled by both sympathetic and parasympathetic activity (Pinsky, 2005; Frazier et al., 2008; Khandoker et al., 2016). Therefore fetal development can be assessed by the ICT, the EDT and the VFT, as well as their interactions which evolve concomitantly with the changes in sympathetic and parasympathetic activities during fetal maturation. As discussed earlier, fetal autonomic brain age can be assessed using FHRV parameters (Van Leeuwen et al., 2003; Hoyer et al., 2013). The results of this current work demonstrated that a new method based on valve intervals outperforms the FHR-based method in estimating the GA although only time and frequency domain parameters and non identical populations were used. Furthermore, the valve interval method was less influenced by arrhythmias, particularly bradycardia, as shown in Table 4. FHR is also influenced by other factors such as behavioral states of the fetus and maternal physiological and psychological conditions, particularly in the second and third trimesters (Mantel et al., 1991; Monk et al., 2000; Ivanov et al., 2009; Marzbanrad et al., 2015b). While FHR might change according to those factors, this may not necessarily affect the estimation of GA by cardiac intervals. Among the cardiac valve intervals which were found contributing to estimation of gestational age, only VFT was correlated with FHR; the correlation of beat-by-beat fetal RR-intervals with ICT, EDT and VFT across 57 fetuses, were (−0.03 ± 0.13), (−0.02 ± 0.10) and (0.50 ± 0.21), respectively.

We note some limitations of the current study; first, the recordings used in this study are short and may not thoroughly represent the FHRV patterns which are used to evaluate the fetal functional brain development (Hoyer et al., 2013). Longer recordings would enable a better comparison of the effectiveness of valve intervals vs. FHRV patterns to assess the development of autonomic control. Further investigation using longer recordings is also recommended to be able to assess the influence of behavioral states and heart rate patterns on the valve intervals. We also acknowledge the recommendation of 5 min ECG for HRV analysis particularly for nonlinear measures. However, short term FHR variability e.g., the variation of beat-to-beat intervals for adjacent 3.75 s-epochs averaged over 1 min has been shown promising for monitoring of fetal development and surveillance of IUGR (Serra et al., 2008, 2009). On the other hand, the fact that most of the nonlinear HRV measures require at last 5 min of heart rate recording, further highlights an advantage of our proposed approach based on cardiac valve intervals, over the FHR-based approaches for GA estimation. Different from FHR-based approaches, the cardiac valve intervals require significantly less recording and measurement time to acquire a reliable estimation of the intervals to assess the fetal development. In clinical practice using ultrasound imaging, the parameters such as VET and PEP can be estimated by averaging over 30 s, and have shown to be well correlated with the gestational age (Mensah-Brown et al., 2010; Cruz-Martinez et al., 2012). This significantly reduces the examination time and the discomfort for the mothers.

The second limitation of our study is that our patient population did not include growth-restricted fetuses. To fully test our proposed technique, it would need to be evaluated on such a population. Although we have not evaluated the cardiac valve intervals for the fetuses with growth issues, this was studied in the literature, for young children (Alkon et al., 2003; van Deutekom et al., 2016). Van Deutekom et al., have shown that both birth weight and conditional height gain were independently associated with PEP, but not with Respiratory Sinus Arrhythmia (RSA).They discussed that as a shorter PEP indicates higher cardiac Sympathetic Nervous System (SNS) activity, this finding suggests that children with low birth weight have increased SNS activity compared with normal-birth-weight children. They associated the increased infant height gain with decreased SNS activity. Similar results specially for female children were observed by Feldt et al. (2011). Although these studies were not on fetuses, it is consistent with our result, showing that it might be extended to the fetal period. Furthermore, our study proposes a new physiological growth estimation method for healthy population, as the first step in identifying fetal development abnormalities. Furthermore, we studied fetal cardiac anomalies and arrhythmias which may confound the evaluation of GA, and obscure the potential detection of growth abnormalities. The third limitation is that the evaluation of the estimated GA using the proposed method for abnormal conditions, shows that the method fails to estimate the GA in presence of some, but not all, heart anomalies. This was particularly noted for the anomalies that affect operation of the valves. From another perspective, these results show that the valve intervals could be used to detect these anomalies that resulted in unrealistic GA estimates or large errors, as also investigated in our recent studies (Marzbanrad et al., 2014b; Khandoker et al., 2016). However, more cases with a large variety of anomalies are required for a more rigorous evaluation of their influence on the estimated GA.

Although in this study we have not considered the fetal gender, it might have an influence on the fetal growth and development. While some studies found no significant differences between male and female FHR during the first and second trimester (Neiger et al., 2004; McKenna et al., 2005), different intrapartum FHR patterns have been reported for two genders (Porter et al., 2016). Another study on term fetuses just before the labor, reported significantly lower values of most linear HRV measures for female fetuses compared to male fetuses, in both IUGR and control groups, as well as higher entropy indices in the control group (Gonçalves et al., 2013). Apart from FHR, the cardiac function was also previously investigated for male and female fetuses and no significant differences in cardiac function were found for different genders, except tricuspid valve E-wave velocity/time velocity integral for the entire tricuspid valve inflow (E/TVI) and pulmonary valve Acceleration Time (AT) (Clur et al., 2011). Based on the literature noted above, further investigations are required to study the gender-specific differences for GA estimation using valve intervals and FHRV. However, we note that in our study, the assumption was made that gender-determining technology would not be available. In other words, we aimed to make a system that could identify IUGR based upon the one dimensional Doppler only. In this context, the inclusion of gender into the algorithm would reduce the applicability of the approach we present.

Results of this study demonstrate that, for acceptable quality DUS and fECG recordings (determined automatically), the average error in GA estimation can be as low as 2.7 weeks, which is comparable to existing expert-driven methods. This proposed approach to GA estimation could be also improved with more accurate methods of quality assessment for 1-D DUS and fECG signals. It should be also noted that the error was obtained by comparing the estimated GA to the CRL estimates as gold standard, while a more accurate way would be to prospectively enroll the study subjects prior to conception and confirm the day of conception. The CRL is however subject to error (95% confidence interval of around 10 days), particularly in case of pathologies or unsuitable positioning (Grange et al., 2000; Callen, 2011). Furthermore the valve intervals have the advantage that they reflect physiological development of the fetus which is not completely aligned with the physical growth of the fetus. As discussed earlier, the sonography methods can be affected by genetic variations, such as the head shape, positioning of the fetus and pathologic conditions. While the error of the ultrasound-based GA predictors increases with gestational age (Caughey et al., 2008; Falatah et al., 2014; Al-Amin et al., 2015), according to our results, the error of the valve-based method does not change with gestational progression. The accuracy of the FHR-based method even increased with advancing gestation. Therefore our proposed physiological measures can be used in second and third trimesters, when the ultrasound imaging measures have a low accuracy and fail to detect abnormal growth, particularly in late gestation (Al-Amin et al., 2015). Overall, the proposed technique can be used as a measure of the physiological development and an adjunct in estimating the GA where ultrasound methods are unavailable or inadequate due to pathologies, unsuitable positioning, lack of skilled ultrasound operators, or other technical issues.

In conclusion, we proposed a novel and automated method for estimation of the GA, which could be performed using low cost, easy to operate devices that requires lower skills/training compared to sonography methods. In contrast to the sonography methods that are based on the physical growth, our proposed method provides assessment of the fetal physiological development. Compared to CRL-based GA estimates as gold standard, our method resulted in 2.7 weeks error for acceptable quality of recordings and also outperformed the GA estimation by FHRV parameters. The GA estimation based on valve intervals was affected by certain heart anomalies which influence the performance of the valves, but less affected by arrhythmias. Remaining errors in estimating the GA could be used as a marker to detect fetal abnormalities. Considering that the valve intervals reflect the autonomic control of the fetal heart, the new method provides automated assessment of the fetal ANS development that could be independent of the fetuses' locations on the growth curve (since our measure reflects neural development and not physical size). As a result the method proposed in this work might provide indications of growth-related issues, such as IUGR, early in pregnancy and potentially lead to early interventions.

## Author contributions

FM, GC, and AK conceived and designed the study. YK performed the clinical experiments and data collection, while YK, AK, and MP carried out the fetal ECG experiments at Tohoku University Hospital. FM, GC, and AK contributed to the development of techniques and analysis tools. The data was analyzed by FM who implemented the signal processing and quality assessment, feature extraction, gestational age estimation, statistical analysis, comparisons and analysis of the results. All authors Participated in the discussion and interpretation of the results. FM, GC, and AK contributed to the writing of the manuscript and all authors reviewed and approved the final manuscript.

### Conflict of interest statement

The authors declare that the research was conducted in the absence of any commercial or financial relationships that could be construed as a potential conflict of interest.
